# Neonatal necrotizing fasciitis with gas gangrene due to peripherally inserted central catheter-related infection

**DOI:** 10.1186/s40792-023-01690-z

**Published:** 2023-06-14

**Authors:** Mitsumasa Okamoto, Yudai Tsuruno, Hiroaki Fukuzawa

**Affiliations:** Department of Pediatric Surgery, Himeji Red Cross Hospital, 1-12-1, Shimoteno, Himeji, Hyogo 670-8540 Japan

**Keywords:** Necrotizing fasciitis, Gas gangrene, Neonate, Peripherally inserted central catheter infection, Dialkyl carbamoyl chloride (DACC)-coated dressing, Povidone-iodine sugar ointment, *Citrobacter koser*i

## Abstract

**Background:**

Necrotizing fasciitis in neonates is a rare and life-threatening infection involving necrosis of the skin, subcutaneous tissues, deep fascia, and sometimes underlying muscles, with a fulminant course and high mortality rate. Necrotizing fasciitis with gas gangrene related to infection of a peripherally inserted central catheter is very rare.

**Case presentation:**

The patient was a full-term female neonate born by vaginal delivery. Following diagnosis of patent ductus arteriosus, indomethacin was administered from a peripherally inserted central catheter for 3 days. Four days after the termination of medical treatment for the patent ductus arteriosus, the patient developed fever and a severely elevated inflammatory response was identified from blood testing. Around the right anterior chest wall, corresponding to the site of the catheter tip, redness was increased and gas crepitus was felt under the skin. Computed tomography revealed emphysema in the anterior chest, in subcutaneous areas and between muscles. Emergency surgical debridement was performed under a diagnosis of necrotizing fasciitis with gas gangrene. With antibiotic treatment, we started to fill the wound with a dialkyl carbamoyl chloride-coated dressing and povidone-iodine sugar ointment after washing with saline once a day. The patient survived and after 3 weeks of treatment with the dressing, the wound had successfully resolved without motor impairments.

**Conclusions:**

In addition to medical treatment and prompt surgical debridement, we used dialkyl carbamoyl chloride-coated dressing and povidone-iodine sugar ointment for antiseptic dressings and successfully treated neonatal necrotizing fasciitis with gas gangrene caused by peripherally inserted central catheter infection with *Citrobacter koseri*.

## Background

Necrotizing fasciitis (NF) in neonates is a rare and life-threatening infection involving necrosis of the skin, subcutaneous tissues, deep fascia, and sometimes underlying muscles. The course of NF is fulminant and the mortality rate is high, so rapid and aggressive surgical debridement is essential for treatment [1, 2]. Determining the strategy for wound care for infant cases of NF with gas gangrene related to peripherally inserted central catheter (PICC) infection is difficult because such cases are also very rare [3].

## Case presentation

**Fig. 1:**
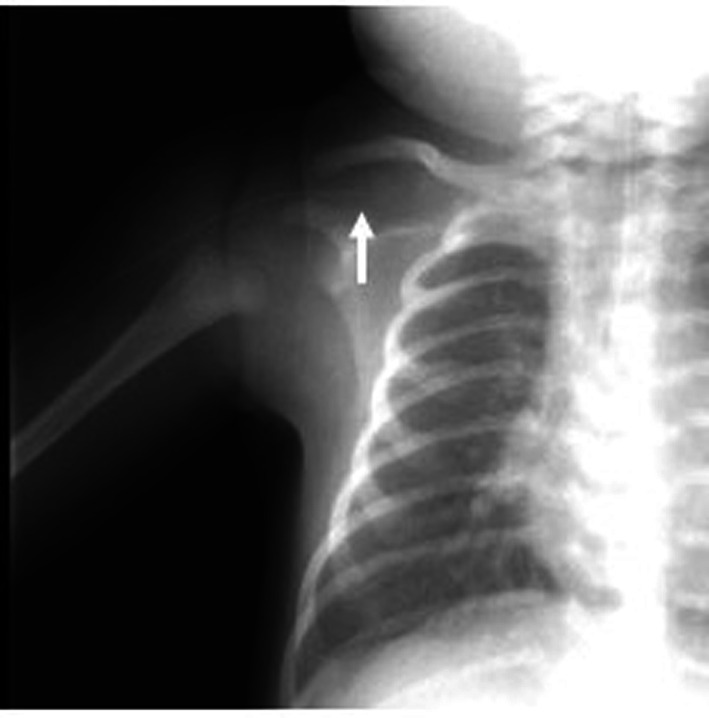
Chest X-ray taken on the day of PICC insertion. Arrow indicates the tip of the catheter. The tip is located in the right subclavian vein

A female neonate was born at a gestational age of 39 weeks 1 day with a birth weight of 2825 g by vaginal delivery. She had been diagnosed with patent ductus arteriosus (PDA). From 5 days old, she received indomethacin for 3 days from a PICC in the neonatal intensive care unit (Fig. 1).

**Fig. 2:**
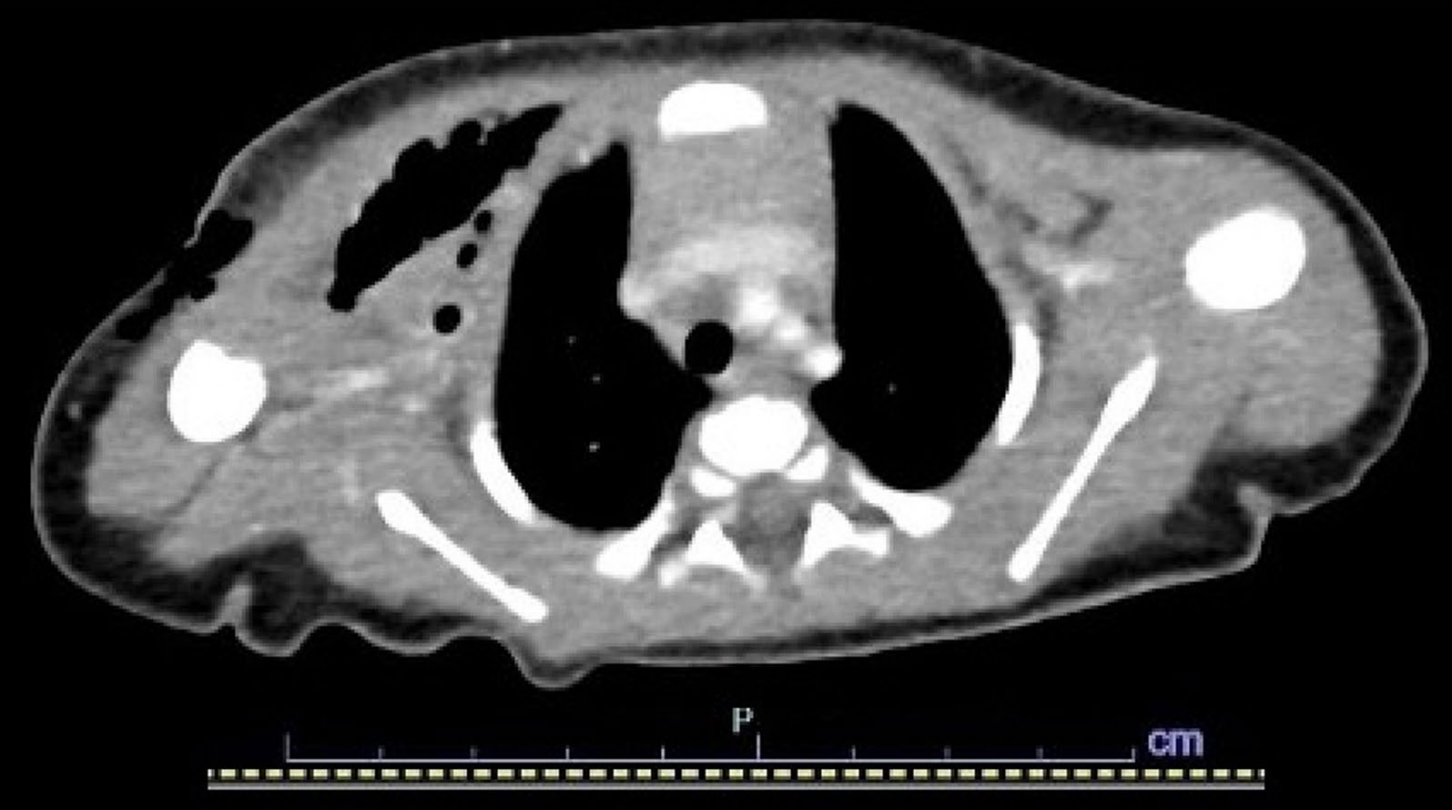
Preoperative contrast-enhanced CT immediately after removal of the PICC. CT shows emphysema in the subcutaneous area and between the muscles of the right anterior chest

Four days after the termination of medical treatment for PDA, fever of 38–39 °C was found and severely elevated inflammatory response was identified from laboratory data, suggesting sepsis (white blood cell count, 61 × 10^2^/μl; platelet count, 8.7 × 10^4^/mm^3^; C-reactive protein, 28.3 mg/dl) was detected. Contrast-enhanced computed tomography (CT) immediately after removal of the PICC revealed emphysema in the anterior chest, in the subcutaneous area and between muscles (Fig. 2).

**Fig. 3:**
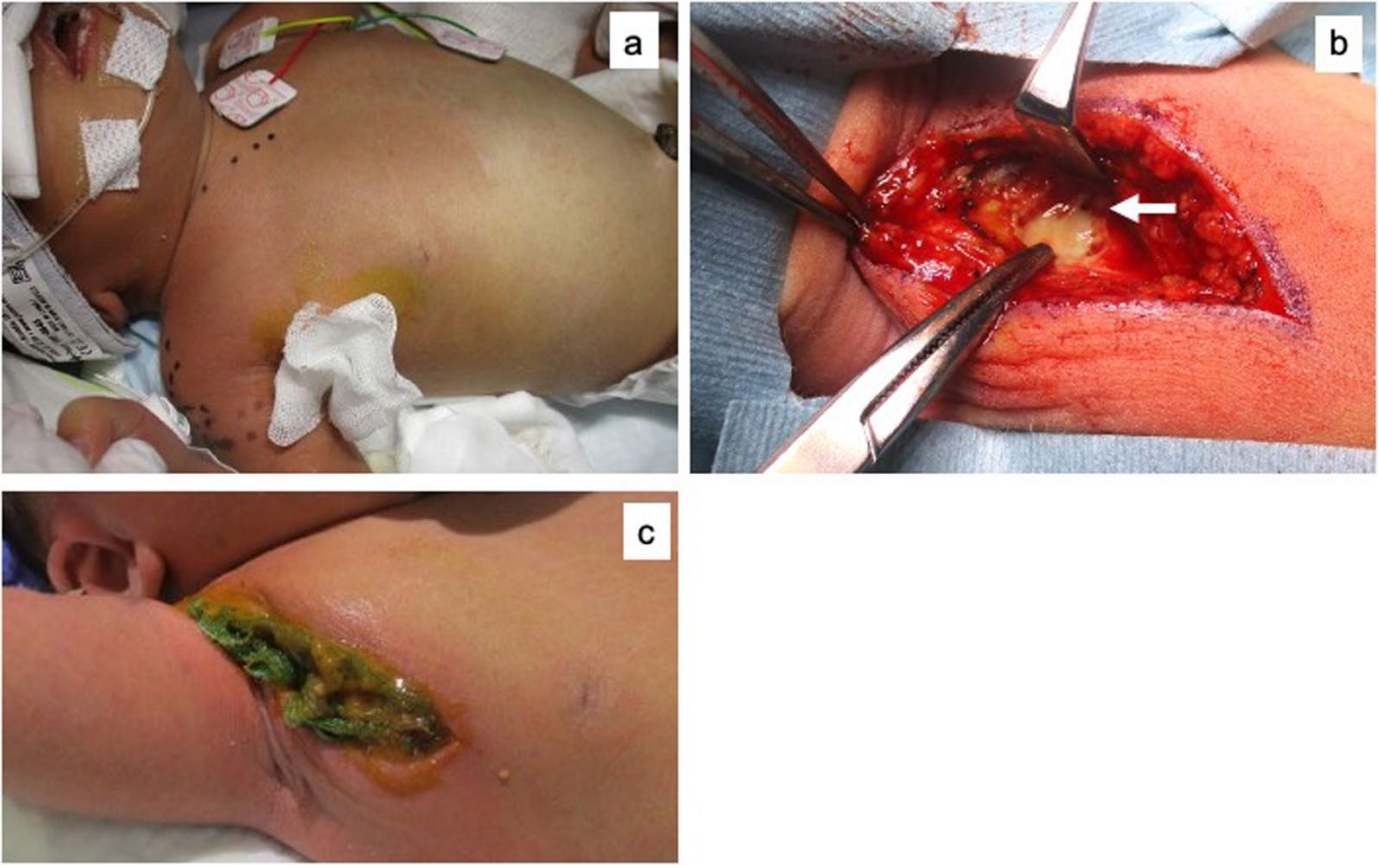
Intraoperative findings. Corresponding to the site of the catheter tip, marked redness of the skin was found (**a**). A pin-hole opening in the emphysema at the necrotic fascia is indicated by an arrow (**b**). Povidone-iodine sugar ointment is wrapped in a DACC-coated dressing and filled into the wound (**c**)

Around the right anterior chest wall, corresponding to the site of the catheter tip, redness was increased and gas crepitus was felt under the skin (Fig. 3a). At 12 days old, emergency surgery was performed under a diagnosis of NF with gas gangrene. A skin incision was made along the outer edge of the pectoralis major muscle from the ribcage to the point where the pectoralis major and biceps brachii muscles intersected. Deep emphysema was released by debridement of the necrotic fascia. No abscess was found inside the necrotic fascia (Fig. 3b). We filled the wound with dialkyl carbamoyl chloride (DACC)-coated dressing and povidone-iodine sugar ointment and changed these daily (Fig. 3c). She rarely cried due to pain during the procedure without medical pain control. The wound healing was rapid and there were no problems such as the residue of infections.

**Fig. 4:**
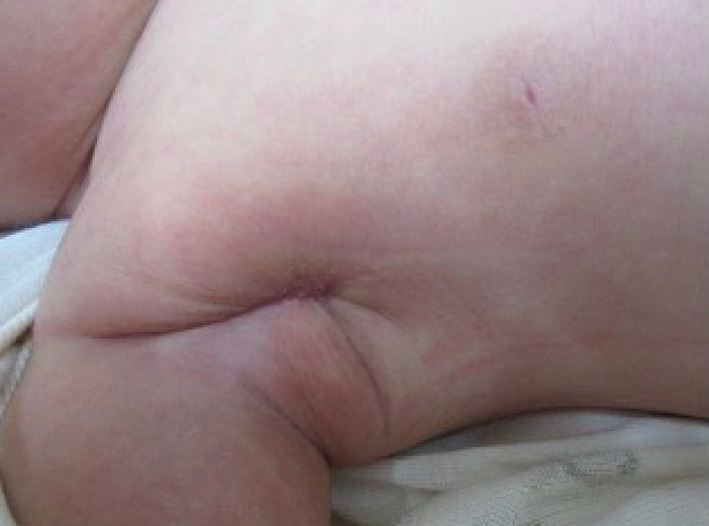
Wound appearance 2 months after surgery. Surgery scar are less noticeable

Carbapenem antibiotics and anti-methicillin-resistant *Staphylococcus aureus* glycopeptide antibiotics were administered until the pathogen was identified, then cephem antibiotics were administered after *Citrobacter koseri* was detected from blood culture and PICC tip culture tests. As a result of 3-week treatment with these dressing materials, the patient recovered well and the wound was successfully cured without motor impairments (Fig. 4).

## Discussion

NF has multiple causes, risk factors, anatomical locations, and pathogenic mechanisms, but all infections in this disease involve a wide range of tissues that can extend from the epidermis to the deep musculature [4]. In adults, NF is common among patients such as those with diabetes, but healthy children can also be affected by initiating factors. Early and aggressive surgical debridement is the most important component of management, given the life-threatening nature of this infection, and delaying operation for more than 24 h reportedly doubles the mortality rate [5].

In neonatal NF, above all, early diagnosis and emergent surgical debridement are essential for patient survival because the mortality rate is high [1, 2]. Magnetic resonance imaging (MRI) is useful for diagnosing NF [6], but contrast-enhanced CT may be suitable for neonatal emergency cases because MRI imaging takes time. In this case, we tried using two materials, DACC-coated dressing and povidone-iodine sugar ointment, for wound care to treat this condition safely and effectively and obtain successful outcomes.

DACC-coated dressings reportedly show strongly hydrophobic properties and wound bacteria also have hydrophobic characteristics, so they become physically bound to the dressing fibers and are subsequently removed from the wound when the dressing is changed without charging the wound with a chemically active agent [7]. It is also described that the use of this product contributes to the improvement of the pain symptoms [7].

No previous reports appear to have described the use of DACC-coated dressings in neonatal NF cases.

Povidone-iodine sugar ointment is widely used for incurable infectious wounds, activating fibroblasts and promoting wound healing. The effectiveness of this ointment in improving wound healing has been proven in clinical surgical wounds [8].

From the results of blood culture testing and PICC tip culture test, we finally concluded the pathogen in this case was *C. koseri*, a Gram-negative rod known as a pathogen in neonatal meningitis. While non-central nervous system infections are rare [9, 10], we should be vigilant for *C. koseri* as a potential causative organism.

## Conclusions

Accurate diagnosis, appropriate antibiotics, prompt and aggressive surgical debridement and effective wound management allowed successful management of this unusual case of neonatal NF with gas gangrene caused by PICC infection.

## Data Availability

Not applicable.

## Abbreviations

CT: Computed tomography
DACC: Dialkyl carbamoyl chloride
MRI: Magnetic resonance imaging
NF: Necrotizing fasciitis
PDA: Patent ductus arteriosus
PICC: Peripherally inserted central catheter
